# Towards Citizen Co-Created Public Service Apps †

**DOI:** 10.3390/s17061265

**Published:** 2017-06-02

**Authors:** Mikel Emaldi, Unai Aguilera, Diego López-de-Ipiña, Jorge Pérez-Velasco

**Affiliations:** 1 DeustoTech - Deusto Foundation, University of Deusto, Avda. Universidades, 24, 48007 Bilbao, Spain; unai.aguilera@deusto.es (U.A.); dipina@deusto.es (D.L.-d.-I.); 2 Tecnalia, eServices, Calle de Velázquez, 64-66, 28001 Madrid, Spain; jpv1983@gmail.com

**Keywords:** open government, open data, open services, open innovation

## Abstract

WeLive project’s main objective is about transforming the current e-government approach by providing a new paradigm based on a new open model oriented towards the design, production and deployment of public services and mobile apps based on the collaboration of different stakeholders. These stakeholders form the quadruple helix, i.e., citizens, private companies, research institutes and public administrations. Through the application of open innovation, open data and open services paradigms, the framework developed within the WeLive project enables the co-creation of urban apps. In this paper, we extend the description of the WeLive platform presented at , plus the preliminary results of the first pilot phase. The two-phase evaluation methodology designed and the evaluation results of first pilot sub-phase are also presented.

## 1. Introduction

The 2016 edition of the EU eGovernment Benchmark Report [1] states that online public services are becoming increasingly accessible across Europe, 81% being now available online. However, deeper analysis of user-centricity, transparency, cross-border mobility and in general quality of use shows that growth is uneven, and a substantial number of EU countries are still lagging behind. This sends a clear signal for acceleration, in order to keep up with private sector pressing needs and citizens’ expectations. While the online availability of services at the EU28+ level reached 81% (+9 percentage points since 2012–2013) and online usability 83% (+4 points since 2012–2013), the ease of using and the speed of using these services online, as perceived by the mystery shoppers, testing online services, advanced poorly, increasing by only one percentage point since the first complete assessment in 2013.

On the other hand, key socioeconomic challenges such as mobility, demographic change, security, environment, employment and many others are being faced by public administrations. The current economic crisis stage has caused the reduction of budgets in the public administration, creating a renewed momentum for the modernization of public administration. In 2015, the EU-28 general government expenditure amounted to 47.3% of GDP [2]. Besides, citizens claim burden reduction and an increase of the efficiency and personalization of the public administration and its services. Citizens want to collaborate in the creation of those government public services, becoming providers of those services instead of being mere consumers. According to [3], an ICT-enabled open and collaborative government is the key to meet public needs in times of tighter budgets, improving the business environment by providing better services to businesses and citizens and adapting service provision to the needs of a more digital economy.

**Figure 1:**
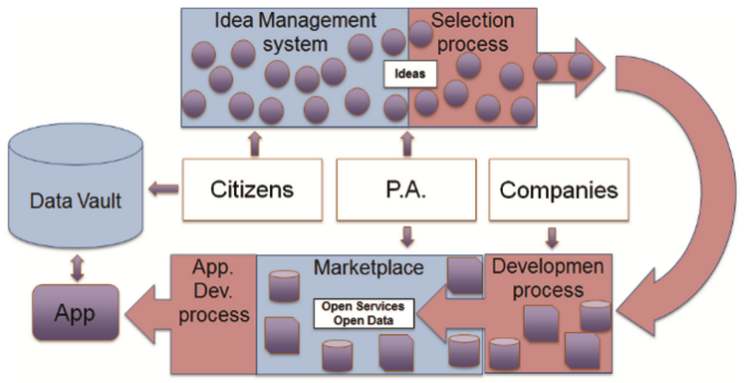
WeLive concept: ideas –> applications –>marketplace.

The main objective of the WeLive project [4] is to transform the current e-government approach by facilitating a more open model of design, production and delivery of public services, based on the collaboration among different stakeholders from the quadruple helix, i.e., citizens, public administrations, private companies and research institutes. Within the WeLive project, a novel ecosystem of tools built on top of the open data, open services and open innovation paradigms and easily deployable in different public administrations have been developed. As shown in Figure 1, the open and collaborative government ICT infrastructure developed for WeLive resembles an assembly line for e-government services. In summary, the WeLive platform offers a set of tools that enable the cooperation among different stakeholders, transforming the needs into ideas, selecting the best ideas and creating the building blocks necessary to build the desired solutions, composing them into mass market apps that are available through the WeLive Marketplace.

This paper has the following structure. Section 5 reviews related work. Section 2 describes the WeLive platform and its key components. Section 3 outlines the WeLive co-creation approach. Section 4 describes the evaluation methodology designed and its application to the pilot in Bilbao. Finally, Section 6 draws some conclusions about results given during the first pilot phase.

## 2. The WeLive Platform

The WeLive platform encompasses a suite of tools that provides governments, citizens, companies and researchers the capability to share and collaborate for promoting innovation. WeLive allows developing the co-creation of innovative ideas (through the open innovation area component) and their implementation as datasets, Building Blocks (BBs) and public service apps, publishing them in the WeLive Marketplace. A building block is a web service providing a concrete functionality that could be combined with other building blocks and datasets, forming an application. There is no restriction about the technologies (programming language, deployment environment, and so on) selected to create services or building blocks inside the WeLive ecosystem, as they fulfill a set of requirements that allow the WeLive platform to expose these services as reusable building blocks.

For those developers who do not want to set up and maintain their own servers, WeLive also provides different hosting environments. Developers can publish their services in the WeLive Marketplace, independently how and where developers expose their services, publishing in the WeLive marketplace and making them available to be used by other developers or end-users.

**Figure 2:**
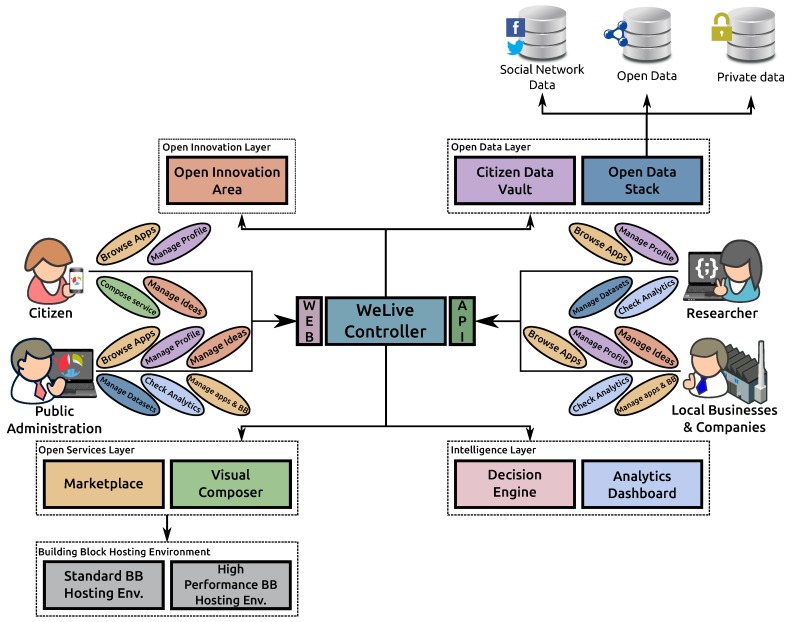
Overall architecture of WeLive platform.

The architecture of the WeLive platform is structured into four layers, as in Figure 2. In this architecture, the Welive Controller central component orchestrates the different requests issued through the WeLive web UI (https://dev.welive.eu/) or the WeLive RESTful API (https://dev.welive.eu/dev/swagger/) to different components:

1. Open Innovation Layer: the objective of this component is to boost collaborative research and development, fulling innovative discovery of novel public services.
2. Open Data Layer: it handles Open Datamanaged by the public administrations plus data provided by the end-users, for example through their smartphones’ apps or social networks. This also manages user personal data in a secure storage named the Citizen Data Vault.
3. Open Services Layer: helps public administrations, companies and citizens to develop new building blocks and public services on top of the Open Data layer. It includes the Marketplace component where WeLive artifacts are published.
4. The Intelligence Layer: through the Decision Engine component, it recommends other related artifacts that could be interesting to the user, based on artifact metadata and user personal data stored in the Citizen Data Vault, increasing the user experience when browsing artifacts (datasets, building blocks and public service apps). The Analytics Dashboard, in conjunction with the Logging Core Building Block, depicts different statistics about the usage of the entire platform.

### 2.1. Open Innovation Layer

The Open Innovation Layer is implemented by the Open Innovation Area component. This component is a co-creation environment where different stakeholders expose their needs, and ideas and possible “solutions” can be tackled by the Public Administration in order to implement them. In this component, different requests meet possible offers. Needs are made public and so highlighted for the community. Different ideas coming from citizens or directly from PAs to satisfy a specific need are suggested. The Open Innovation Area offers tools for: (a) eliciting, analyzing and improving ideas proposed by different stakeholders; (b) allowing different stakeholders to vote and select the more suitable ideas for a need; (c) allowing companies to offer a technical solution to selected ideas, being funded by interested groups of citizens or directly by the public administration.

It implements the following procedural steps to carry out Open Innovationwithin a city through the co-creation of public services:

(a) A citizen or a business representative can publish a need arising in her/his city, in order to find a solution collaboratively for the “development” of the territory. Based on this need or a set of related needs, the municipality can create a challenge. The aim of a challenge is to collect ideas from citizens in order to solve an specific issue.
(b) Citizens can provide new ideas to the challenge. These ideas can be co-defined through social and collaboration features like comments and ratings.
(c) The municipality can select the idea which most fits with the objective of the challenge, proceeding to the refinement phase. The aim of this phase is to concrete the requisites of the possible solution in order to transform the idea into a building block, dataset or public service app.
(d) At the refinement phase, citizens could contribute with their thoughts about how the selected idea could be transformed into a suitable solution, providing their own experience on the diverse fields needed to fulfil a solution (co-experience). Once all of the requirements are fit, the idea proceeds to the implementation phase.
(e) At the implementation phase, citizens could compose a public service app using the Visual Composer, a future WeLive component that will enable non-technical people to mash-up building blocks and datasets in order to create new public service apps; or developers could develop an app or a building block using tools of their choice (co-development).
(f) At last, the resulting implementation could be published in the WeLive Marketplace, making it available to all stakeholders coming to the city (co-delivery).

### 2.2. Open Data Layer

**Figure 3:**
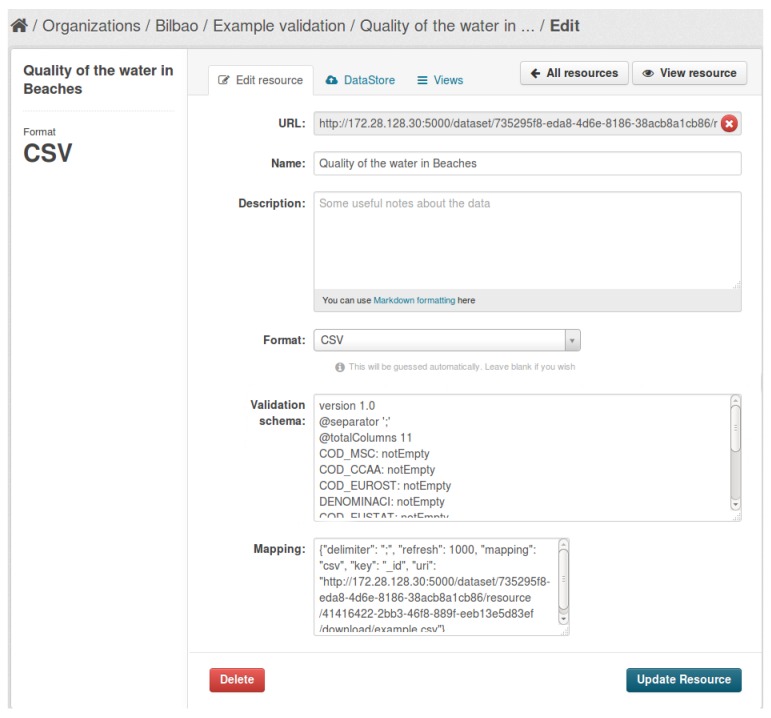
The resource edition form with a CSV schema and a mapping for the Query Mapper.

**Figure 4:**
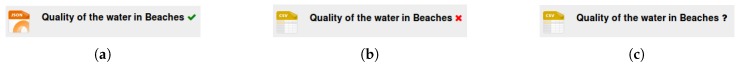
The possible outputs from the validation module are a successful validation (**a**), a failed validation (**b**) and the validation schema is missing or it is not valid (**c**).

The Open Data Layer manages the Broad Data [5] gathered within a city, coming from different heterogeneous sources such as Open Government Data, citizen supplied through social networks or mobile applications, or private sector data publicly available. For that, it provides the Open Data Stack and the Citizen Data Vault components. The Citizen Data Vault keeps user’s personal data, preferences and profile details in a safety vault where they are accessed only under user control. Built on top of the popular data management system CKAN –Comprehensive Knowledge Archive Network– (https://ckan.org/), the Open Data Stack serves to address the main steps of the lifecycle of broad data management in a Smart City [6]: data capture, curation, storage, publication and exploitation. The ODS functionality consists of the following internal modules:

- Data source management: this module provides the basic functionality to perform CRUD operations with datasets and its associated resources. The component provides a user interface to facilitate the management of this data. It is mainly the core CKAN functionality with some new developments for fitting WeLive’s requirements.
- Rating system: this module manages the ratings of datasets and its associated resources that can be provided by Open Data Stack users. Users can provide its rating for a dataset, and the system will compute the aggregated rating for all of the users. In conjunction with CKAN’s dataset commenting system, it could provide an indicator about the quality of the datasets according to users’ opinions.
- Validation: this module allows validating the data from a resource using different validation schemas like the CSV schema (http://digital-preservation.github.io/csv-schema/csv-schema-1.0.html), the XML schema (http://www.w3schools.com/xml/schema_intro.asp) or the JSON schema (http://json-schema.org/) and RDF [7] resources based on the ontologies used. For validating resources, the creator of the resource can specify the proper schema in the resource edition form (Figure 3), and the ODS states if the resource fits this schema, as can be seen at Figure 4.
- Harvester: the harvesting module allows gathering metadata from external data sources and including them in the ODS. Through the web interface, a user can configure a harvesting source, and this source is checked periodically looking for new data. At this moment, the following sources can be harvested: external CKAN instances, DCAT catalogs, web pages (using regular expressions or XPath –XML Path Language– expressions) and the Twitter social network. As the harvesting modules expose a set of interfaces, developers could implement new plugins for harvesting data from other social networks and non-web sources. New datasets and resources are created inside the ODS from metadata gathered from these sources, but the created resources link to the original location of the data.
- Query Mapper: created as part of the IES Cities European project [8], the Query Mapper allows performing SQL queries over data resources existing in the ODS, returning results on JSON format. For enabling this functionality, the resource has to be mapped through the proper JSON mapping, as can be seen in Figure 3. In the case of JSON, CSV and RDF data, if the user does not provide a proper mapping for a given resource, the ODS tries to create a suitable one automatically. At this moment, supported file formats are CSV, JSON, RDF and relational databases. In the future, it will be suitable to enrich this component enabling the interoperation with widely-used No-SQL databases.
- API: this component provides a programmatic access interface to all of the functionality of the ODS, which is useful for the integration of this component with other WeLive project components and the usage of the ODS functionality by developers creating applications. For easing the understanding of this API by the developers, a Swagger (http://swagger.io/) interface is deployed within the API.

### 2.3. Open Services Layer

**Figure 5:**
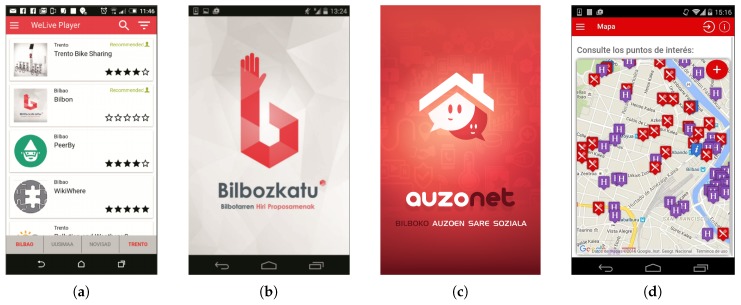
WeLive Player (**a**), Bilbozkatu (**b**), Auzonet (**c**) and BilbOn (**d**).

The Open Services Layer is for centralizing the management of the main artifacts managed by WeLive (public service apps, building blocks and datasets). This layer comprises the following components:

- The WeLive Marketplace is the repository where different apps, building blocks and datasets are published. It is the core component to exploit the economic potential brought forward by WeLive. There, WeLive artifacts can be browsed, selected and exploited by different stakeholders. In the future, the functionality required to enable purchasing of artifacts as SaaS and DaaS [9] will be implemented.
- Hosting environments: The WeLive project offers different hosting environments in which developers can host the business logic back-end of the WeLive artifacts. Such hosting might be done in one of the environments provided by WeLive or, alternatively, at a third party, cloud-based IaaS or PaaS, as long as artifacts hosted there are WeLive Open Services framework compliant. The project currently offers two different hosting environments: (a) a CloudFoundry-based hosting environment as a general-purpose solution to host web apps in the cloud; and (b) a hosting environment operated within the Cloud’N’Sci.fi (https://cloudnsci.fi/) marketplace for hosting computationally-complex and/or commercialized BBs. If the WeLive provided hosting environments do not fit the user’s need, she/he can publish her/his artifact in her/his own hosting environment.
  Independent of where an artifact is published, they must provide a set of standard descriptions depicting both general-purpose information (e.g., providers and authors, license info, classification) and technical information (e.g., the supported protocols and formats, security constraints). These specifications allow the Visual Composer to “understand” the functionality of building blocks and the content of datasets, enabling the construction of mash-ups using artifacts hosted in the WeLive Marketplace. The last update of the WeLive Marketplace includes a wizard that eases the task of generating these WADL and USDL files and publishing the artifact in the Marketplace.
- WeLive Player: an app to access the city service ecosystem (see Figure 5a). It permits searching and discovering the public service apps of the city, filtered through the user’s context, profile and preferences, with the help of the Decision Engine.

The Open Services Layer provides a set of Core Building Blocks. These Core Building Blocks expose functionalities to be used by both by the platform itself and by new building blocks. The most remarkable ones are:

- Authentication and Authorization Control (AAC) BB provides the functionality of the centralized user authentication and authorization across apps and tools in WeLive platform. Thanks to this core BB, users can reuse the same credentials fore each component or app from WeLive platform.
- Logging BB allows for tracing information regarding the platform-level and application-level events. These logs are used by the Analytics Dashboard to depict statistics about the usage of components and apps from WeLive platform.

### 2.4. Intelligence Layer

This layer encompasses the Analytics Dashboard and Decision Engine components. The Analytics Dashboard allows gaining insights and generating visualizations about the usage of a WeLive deployment in a council, e.g., how apps are being used, what new apps are being required or what services each stereotypical user profile demands or uses most are some of the possible indicators depicted by this component.

On the other hand, the Decision Engine brings together the final user interests, needs and wants with the ecosystem of public services (apps) in the WeLive Marketplace. It includes a set of recommenders, allowing other tools from WeLive framework offering a better user experience. Concretely, the Decision Engine is able to make recommendations among different artifacts, allowing users to reach to artifacts of their interest. In the same way thanks to the anonymous profiles collected in the Citizen Data Vault, the Decision Engine is able to recommend apps of her/his interest to a given user. Regarding the Open Innovation Area, the Decision Engine recommends collaborators for developing an idea. In addition, given an idea, Decision Engine will recommend other related ideas.

## 3. WeLive Co-Creation Approach

The WeLive project bases its service co-creation and innovation approach on the paradigm known as the “Quadruple Helix” [10], where the fourth helix is the citizen, who closely collaborates with industry, government and academia to co-create value-added public services.

The WeLive platform enables stakeholders to participate by contributing with new services, building blocks and dataset requests, by giving place to new data and apps and even by co-funding novel public app ideas, consuming these generated apps through the WeLive Marketplace.

During the first 24 months of the project, there were two different phases in which the co-creation process has been applied. At the first one, known as “Stakeholders Consultation Process” and executed during the early stages of the project, the co-creation process has been developed without the usage of the Open Innovation Area tool, which has been used during the second phase, named as the first pilot phase and executed at the second half of 2016. From May 2017, the second pilot phase is going to take place. Despite the WeLive project spanning along three cities (Trento (Italy), Novi Sad (Serbia) and Bilbao (Spain)) and one region (Helsinki-Uusimaa in Finland), this paper is focused on describing the co-creation process developed in Bilbao. In this section, the tools and techniques used during the co-creation processes are explained, while results given from these processes are presented in Section 4.

### 3.1. Stakeholders Consultation Process in Bilbao

**Figure 6:**
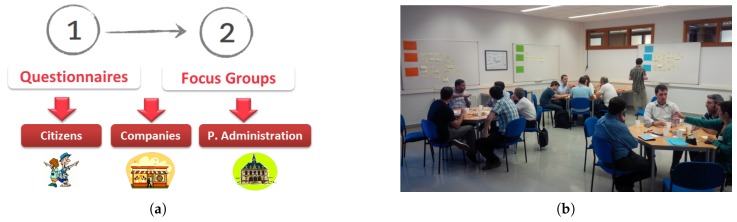
Stakeholder consultation process in Bilbao (**a**) and participants from the citizens’ focus group (**b**).

The first step consisted of distributing questionnaires among different stakeholders (citizens, companies and public administrations) by e-mail (Figure 6a). These questionnaires had both open-ended and closed-ended questions. In the closed-ended questions, the participants were required to choose among one of the given answers, while in open-ended questions, participants could write free text in response to the questions. Among closed-ended category questions, questions about the age-range of participants, occupation, where they live/is the company established/public administration belongs, usage of ICT technologies and digital services, their interests in different public sectors, open data published by public administrations and services or resources freely offered by companies can be found. On the other hand, questions about personal and contact information, used and wanted digital services, digital services that can be useful for close people, data to be opened by companies, open data wanted by companies, open data gathered by public administrations and its usefulness, in which projects related to open data are public administration participating and open data resources to be published by public administrations, could be freely answered by the participant. Once questionnaires were responded, Bilbao’s task force has analyzed given answers. This analysis revealed that surveyed stakeholders were more interested in the areas of traffic, culture, health and tourism.

For going deeper into service definition for these areas of interest, at the second step, three focus groups [11] were carried out, one for each stakeholder (citizens, companies and public administrations) and inviting the participants of questionnaires that have provided their contact information (Figure 6b).

The focus groups have been organized as follows. At first, participants were divided into different groups, one regarding health and the environment, another one about transparency and citizen participation and the last one about how to best interact with services of the city and the city in general terms. After the reception of assistants, a brief description of the WeLive project and its objectives was done by the moderators of the meeting. Next, the objectives of the focus group and the methodology to be implemented in the focus group were presented. Once the operation of the workshop was explained, a warm-up exercise was guided by moderators. In this exercise, the participants of each group were encouraged to draw whatever they wanted inside circles drawn in a folio. After this warm-up exercise, the brainstorming started. Each group, guided by a moderator, was formed by four or five persons and was focused on one of the main areas of interest. For each area of interest, a table describing facts and ideas was made. The facts referred to the problems that participants of the group had in their daily life, related to a concerned topic. On the other hand, the ideas represented the solutions suggested by participants for solving each problem. Even so, facts and ideas could be independent from each other: a participant could propose a problem without figuring out a solution or a new service that does not solve any concrete problem. From these empty tables, participants filled them with post-its representing their facts and ideas. This stage lasted about 25 min.

After filling the tables, participants went through all groups voting on the best facts and ideas. Each participant had the opportunity to vote up to the third part of the proposed facts and ideas. From most voted ideas, participants had modeled different services in the form of use cases. Once these use cases were described, participants explained the proposed services to the rest of the participants. After explaining these services, participants voted for choosing the best services, finalizing the workshop with a discussion about most voted services. It is worth mentioning that all assistants to these workshops signed a declaration of consent, coping with the ethical compliance procedures established in WeLive.

### 3.2. Population of WeLive Environment for Bilbao

For the first phase of the pilot, a set of services was selected among the ideas gathered through the focus groups, questionnaires and internal meetings. These services made use of a set of specific building blocks for the city of Bilbao. Concretely, three applications and six building blocks were developed. The applications are the following ones:

- Bilbozkatu (neighborhood citizen voting): “Bilbozkatu” (Figure 5b) allows citizens launching and voting proposals in response to different issues or questions within the city. This service is a dashboard for easing the understanding of different collectives of inhabitants living in several neighborhoods of Bilbao and for promoting activities and actions that best fit in each zone. Through their contributions and votes, citizens have the opportunity to participate, collaborate and co-decide how they want their neighborhood to be. It is certainly an application of digital citizen polling, which also meets a real social need. Civil servants, through this service, are able to really know what the main needs and preferences of Bilbao’s citizens are. This information might be used to ease the process of decision making related to new investments.
- Auzonet: this app (Figure 5c) enables a neighbors’ social network to provide solutions to the inhabitants in those neighborhoods, based on proximity and confidence. Users post something they want to borrow; neighbors willing to lend things get a push notification to which they can respond in a single touch; and the borrowing process is enabled. Citizens are able to locate on a map other citizens’ needs. That way, this app may encourage collaboration among neighbors. The app also lets the users provide feedback about the service, so future users can select one service or another based on this feedback.
- BilbOn (WikiWhere): the third service for Bilbao is an app (Figure 5d) which aims to provide information about public utilities’ location (e.g., toilettes, mailboxes, containers, bikes). It offers a public catalog of urban points of interests, geo-located news, warnings and other geo-tagged information in order to better know the city. This service also allows tourists and citizens to organize their tour and visit the city following an itinerary, allowing them to enrich each point of interest with their own content. Besides, citizens can collaborate in the creation of new POIs by sending new information to the service.

Among developed building blocks, we can find the following ones for Bilbao:

- Users feedback: this building block adds the “comment” and rating functionality into the services. This means that this building block allows users to provide feedback, opinions and comments about anything and to rate them from 1–5; for example: feedback about other users or feedback about resources of the city. It is used by all of the apps in the first pilot phase.
- Users ranking: for those public services that require making a matching among different users according to users’ rating and preferences. This building block shows the ranking of each user (from the evaluation/rating made by the rest of users) and provides information about how reliable the user is. This enables end-users to select those with a better ranking. The building block offers mechanisms to rate a given user’s service and to retrieve the rankings associated to users participating within a service. This BB is used by Auzonet.
- Near Point Finder: this building block, given a geodesic position (latitude, longitude) and a set of target points, returns the walking distance to the target points, simultaneously reverse geocoding them, i.e., transforming the source and target points’ locations into human understandable addresses and place names. It is a useful building block for some other urban apps like Auzonet.
- Image uploader: this building block allows uploading and sharing photos from apps and other services easily. At this moment, images are being stored on Flickr (https://www.flickr.com/). During the first pilot phase, this is being used by Auzonet.
- In-app questionnaires: this building block allows apps offering the in-app questionnaires used to evaluate the quality of the public services. This building block has a set of RESTful API methods to allow retrieving the questions and submitting the answers easily from the app.
- OSM elements: This building block enable applications retrieving information about the POIs that are available in a zone specified by the mobile service application.

In addition to building blocks and public service apps, datasets from the Bilbao Open Data (https://www.bilbao.eus/opendata/es/inicio) platform have been gathered by the ODS using its harvester module (Section 2.2), in conjunction with some datasets from Open Data Euskadi (http://opendata.euskadi.eus/w79-home/eu). A total of 164 datasets are currently available in the ODS component as seen in https://dev.welive.eu/ods/dataset?organization=bilbao-city-council.

### 3.3. First Pilot Phase

**Figure 7:**
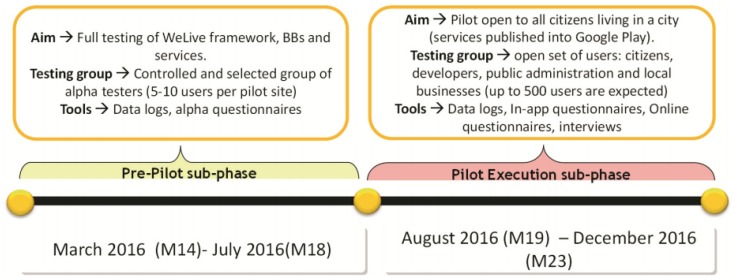
First pilot phase evaluation sub-phases, aim, testing groups and tools used.

The first pilot phase has been split into two sub-phases: the pre-pilot sub-phase and the pilot execution sub-phase (see Figure 7). During the pre-pilot sub-phase, the dissemination material and the preparation of the pilot was done. During this phase, the WeLive platform only was evaluated by a restricted set of users, with the objective of detecting bugs and major issues.

Activities planned for the pre-pilot sub-phase were the following ones:

- Communication activities: these activities include the creation awareness material about WeLive platform and Bilbao services and the selection of 5–10 alpha testers to evaluate the platform.
- Training: the objective of the training workshop is to train alpha testers on how to use the WeLive platform.
- Support: this activity implies giving support to users involved in the pre-pilot sub-phase by email.
- External release: the objective of this activity is to release the tools of WeLive platform and apps, making them available to the overall public.

For the pilot execution sub-phase, the following activities were planned:

- Communication activities: extending communication activities tackled at the pre-pilot sub-phase; for this sub-phase, an intensive dissemination of the WeLive project was planned, approaching different target groups through municipal web portals and social networks. Four workshops were celebrated, concretely a workshop explaining the usage of the platform and apps, one with entrepreneurs, another one with citizens and an idea contest promoted by the Open Data Service Area Mayor’s Office.
- Support activities: provide an email to receive problems with the platform, and publish a FAQ and a usage manual.
- Execution activities: the objective of execution activities is to re-deploy components from WeLive platform and apps after pre-pilot sub-phase conclusion.
- Monitoring and evaluation activities: these activities consist of analyzing logs stored at the Logging Core Building Block and surveys collected during the workshops in order to extract conclusions from the first pilot phase.
- Execution (reaction) activities: the objective of these execution activities is to address possible issues detected during the pilot phase and to intensify campaigns if objectives are not being met.

In Section 4, results from the execution of these planned activities are shown.

## 4. Evaluation

In this section, the results of the evaluation done during the first pilot phase are shown. Instruments available to evaluate this pilot phase were alpha testing, data logging and KPI monitoring, questionnaires, in-app questionnaires and user support. In Section 4.1, the feedback received by pilot participants and members of the consortium about the evidence collection procedures is analyzed. Later, in Section 4.2, the discussion about the collected evidence is tackled.

### 4.1. Evidence Collection Procedures

This section includes a reflection on the procedures applied during the Pilot Phase I for the collection of the evidence used for the monitoring of the pilot’s execution. These reflections serve to improve the data acquisition procedure during the Pilot Phase II of the project. The following sections cover the different aspects of the evidence extraction procedure focusing on the following questions, which have been answered by the different partners of the WeLive project (technical and non-technical) that have used the procedures and data to monitor their pilots:

(a) What has worked well?
(b) What has not worked well?
(c) What are the lessons learned for the Pilot Phase II?

The responses to each of these questions, for the different evidence collection sources, are summarized in the following tables.

#### 4.1.1. Alpha Testing

During the early test state of the platform and the services, at the pre-pilot sub-phase, a simple questionnaire was created in order to gather initial responses about their acceptance, detect technical problems and allow participants to make suggestions. Participants in the alpha testing were taken from a reduced set of users belonging to the different organizations taking part into the project. The alpha questionnaire contained the following questions:

- Name, email and age of the participant: used for further questioning if required and demographic purposes.
- Name of the platform component or application being tested: the participant could provide feedback about different components and applications.
- Feedback about the reported component or application: the text feedback provided by the users was analyzed to detect how to improve the components for the Pilot Phase I.

In addition to the information provided by the users through the alpha testing questionnaires, the participants also provided feedback about the applications and platform components during the events.

Conclusions from alpha testing for the Bilbao pilot arise that although the sessions were very valuable to detect problems that arise for real users when they interact with the platform or the applications, more non-technical users where expected to detect non-technical and usability issues, as technical users are familiarized with these kind of web tools. Although users were sometimes reluctant to provide long textual descriptions of their problems and suggestions after testing the components and applications, alpha questionnaires enabled collecting the feedback of the users about the different applications and components they were testing. The alpha testing was carried through a cross-testing process where we assigned teams’ city task forces to evaluate the apps from other cities. The results of the alpha testing were used to improve the different components of the platform and the services developed for the Pilot Phase I before the actual pilot execution sub-phase started.

#### 4.1.2. Data Logging and KPI Monitoring

The timeline of the pilots envisaged for the project is as follows. Pilot phase I took place from March 2016–December 2016—a ten month time span. Pilot Phase II is taking place from April 2017–December 2017—a nine month time span. Besides, both pilot phases were sub-divided into pre-pilot launching and pilot execution launching phases. Given the long evaluation period in both phases, it was deemed convenient to establish a monthly period to collect all of the KPIs associated with the platform tools and WeLive artifacts’ usage. Such a collection has been followed monthly by an analysis of the progress in the fulfillment of the KPIs so that actions can be taken quickly to correct mismatches in their fulfillment. As a result, the project is continuously monitoring and reacting to the progress in the achievement of its indicators. For instance, if the usage of some of the generated apps is low, further engagement activities may be carried out in order to ensure that a wider audience gets access to our tools and artifacts.

A set of indicators has been established as a way to measure the progress of the project towards the achievement of its six core objectives:

1. Societal Objective 1, i.e., SO1, to promote the economic growth and job creation with added-value vertical apps and datasets.
2. SO2, to increase transparency and trust in public administrations through new datasets and apps.
3. Technological Objective 3, i.e., TO3, to provide holistic support for the Open Innovation process of public services.
4. TO4, to streamline the exploitation of Open Data from public services.
5. TO5, to democratize creation of novel public services.
6. TO6, to enable personalization and analytics of public services.

The resulting set of KPIs to measure through indicators the achievement of the above WeLive objectives has been classified into the following categories:

KPI1, Open Innovation Area as a promoter of public service apps.
KPI2, Open Services Framework as facilitator of public service app creation.
KPI3, Stakeholder Behavior Change towards e-Government through WeLive.
KPI4, Age and gender distribution of the registered users.

Furthermore, for each of the developed public services, some service specific KPIs have been created. For instance, for the app BilbOn, which enables crowdsourcing information about POIs in the city of Bilbao, one of the defined KPIs is the number of new POIs added.

All of the actions that the users perform on each of the components of the WeLive platform produce the log entries that are stored and, therefore, can be later processed to extract the KPIs values defined for the project. During the Pilot Phase I, trial coordinators had two different mechanisms for the visualization and periodic analysis of the KPI values obtained from processing the recorded logs. The first of them is the Analytical Dashboard (ADS) component, which provides graphical representations of the platform and application KPIs for each of the city pilots. The ADS enables the trial coordinators from each city pilot to easily visualize the status of the general KPIs and the specific ones defined for their pilots. One of the main concerns about the ADS expressed by users, and to be fixed for the second pilot phase, was about the lack of an historical view of the collected data, which could be useful to inspect the trends in the measured KPIs.

The ADS was fed by logs collected by the Logging Common Building block. This BB exposes an API that allows other components and apps registering events in order to generate statistics. This component was very useful during the first pilot phase, but the responsibility of properly logging the events leverages on components and apps developers, and they could forget, or misspell, the required log messages.

On the other hand, the trial coordinators must periodically (i.e., monthly) extract the KPIs values to analyze the trends in the KPIs. This periodic extraction procedure is performed through an internal tool that enables downloading each pilot data in a CSV file that can be stored and processed, if required, with other external tools to perform further analysis. As it was a script, the usage of this tool required minimal technical knowledge. This was fixed by the development of a web page that allows users with non-technical skills downloading a CSV file with each pilot data.

#### 4.1.3. Questionnaires

Questionnaires allow gathering the qualitative perception that the participants of the pilot had about the different components of the platform and the developed services during the Pilot Phase I. Particularly, there are two types of questionnaires that were used in the different events organized during the Pilot Phase I.

The first of them is the demographic questionnaire, which is oriented toward the gathering of information about the characteristics of the attendants to the events. The output of this questionnaires can be later used to infer how the platform and the application are accepted by the different demographic sectors that have attended the events and to detect the bias in the selection of participants and in the extracted conclusions for Pilot Phase I.

On the other hand, in addition to the demographic data gathering, Pilot Phase I used other questionnaires oriented toward obtaining specific information about the platform and its perception from the different stakeholders that took part in the events: citizen, companies and public sectors.

The questionnaires were available in printed and on-line formats and translated by each city pilot to their local language in order to adapt to the participants during each pilot event. The usage of paper questionnaires has the disadvantage that they require processing a high quantity of information in order to extract conclusions. In order to ease the analysis and generation of survey data, some spreadsheet templates were created during the Pilot Phase I.

#### 4.1.4. In-App Questionnaire

The in-app questionnaire was oriented toward extracting information about the user perception and usage of each service developed by each city task force for Pilot Phase I. This questionnaire was shown to the users during the interaction with the mobile applications, and it contained five rating-based questions and one free text question allowing users to provide further feedback about the application. In addition, the users can provide their email address for further contact and questioning.

The questions contained in the in-app questionnaire are the following:

- Do you like the app design? (1–5 stars)
- Was this app easy-to-use? (1–5 stars)
- Was this app useful for you? (1–5 stars)
- Does this app increase your sense of community? (1–5 stars)
- How likely is that you would recommend this app to a friend? (1–10 stars)
- Provide feedback about the app. (Free text)

The in-app questionnaire supports translation to multiple languages, meaning that users receive the questions in the language configured in their mobile devices. Once the users filled the responses to the questions, the data were stored in a database, and they were later retrieved by the trial coordinators of each city pilot in CSV format.

Trial coordinators of each pilot city were required to periodically inspect and analyze the responses provided by the users through the in-app questionnaire, particularly the free text responses that require human intervention to be understand and processed. In addition, the in-app questionnaires produced different KPIs that can be accessed through the Analytic Dashboard (ADS) or the monthly KPI extraction mechanism.

Although the question set selected as the in-app questionnaire enabled capturing basic and general feedback about each application tested during the Pilot Phase I, the number of filled in-app questionnaires was low for some applications. In order to solve this issue, a Survey Building Block was been developed for the second pilot phase, allowing app developers to define their own questions and possible responses.

#### 4.1.5. User Support

In addition to all of the previous forms of data gathering, each city pilot defined a user support mechanism that allows event participants, and more generally, platform and application users, to ask their questions and problems about the platform usage. The questions and problems obtained through this channel were very valuable to each city project and to the whole project to identify the common misconceptions about the platform during its general use and to generate specific support documentation and extend the FAQ for the platform and services.

The initial organization of the documentation generated for the first pilot phase was chaotic for non-technical users. In order to make this documentation clearer to all users, it was simplified for Pilot Phase II by creating three new documents on the different group of users: citizens, developers and administrators of the platform. On the other hand, the maintenance of the documentation and FAQ by consortium requires some technical knowledge. In order to ease this task, the Pootle (http://pootle.translatehouse.org) translation manager tool was deployed, providing a web interface for translating the documentation and FAQ.

### 4.2. Discussion on Collected Evidence

This section provides a summary of the main outcomes and problems detected after processing the data from each evidence source during Pilot Phase I of the WeLive project.

#### 4.2.1. Activities Summary

This section summarizes the activities that were organized during Pilot Phase I in Bilbao. During Pilot Phase I, the Bilbao city task force has organized a total of 14 events (four “Inform me” events, nine “Guide me” events and one “Feedback event me” event) distributed along the six months of the pilot phase. The following points summarize the main outcomes of the different events organized during Pilot Phase I in Bilbao:

- Activities of relationship with stakeholders: the Bilbao pilot has developed a series of activities with a significant sample of the identified stakeholders of the WeLive project: Public Administration, citizens, entrepreneurs, companies in the digital sector, etc. This sample was small, but very representative and sufficient sample to validate certain concepts and Essential practices for this phase of the project are the formulation of the challenges, the Contests of Ideasand the selection of ideas for new public services.
- Activity with public administration: with respect to the Public Administration, we have developed information and presentation actions on the WeLive platform and the three Developed Services with the directors and management teams of the City’s main departments, such as the Mayor’s Office, Economic Development, Citizen Participation and Attention, Mobility and Sustainability, Works Management and Public Space, Tourism Offices, etc., among others.
- General results achieved from the work with the Public Administration: the main results of this activity of Workshops with the administration teams have been the work of formulating different challenges expressed by most of the Areas around which to raise the impulse of processes of collaboration with citizens and interest groups and the organization of two Contests of Ideas were celebrated with the collaboration of two of the areas (Economic Development and Youth) within the framework of this pilot.
- Experience with challenges: from these challenges, those formulated by the Economic Development and Youth and Sport Areas have been selected as the most suitable for this pilot because they have very stable relations with different stakeholders implied in the development of the city. In addition, these areas had scheduled initiatives coinciding with the project WeLive.
- Activity developed with sectors of the citizenship: collectives of start-ups, digital creators, young people, etc.
- Focus on preferred collectives: the focus on the pilot has been developed with a sample of groups of entrepreneurs, social innovators, digital creators, etc., with a general profile of young people and with a broad relationship with technology and processes of innovation; people are very interested in continuing working on the next months and specifically for the second pilot in 2017.
- Experiences with ideas contests: two idea contests have been promoted during Pilot Phase I, directed mainly to the segments of entrepreneurs, groups of digital creation and young people. These challenges have been focused on topics that are of special interest for the pilot city. The first of the challenges aimed to identify digital solutions: apps, webs, etc., to boost communication between these sectors of entrepreneurs, digital creators, start-ups and for the search of synergies and collaboration in their relations with the administration. The second challenge was related to the search for solutions that serve to publish a map of initiatives in the city in the areas of culture, creation and sports and to promote communication between these groups and the administration in order to promote a city that is more innovative, creative and geared towards attracting talent.
- Organization of contests in events: contests have been launched in every relevant events for elected collectives such as Free Software Congresses, and in centers and facilities of the city associated with the activity of these groups.
- Ideas obtained in the ideas contests: the majority of the ideas provided by the participants are aligned with council expectation. Now is the period for analyzing their transformation into future new public services.
- Activity with ICT companies and focus on local references of ICT companies: we have worked with a group of people belonging to the most important local ICT companies and referents in the development of digital solutions, experiences with Open Data dynamics and participation in social networks.
- Results of activities with ICT companies: during the pilot, relations and work sessions have been maintained with people from local ICT companies who will continue with the second pilot to study their collaboration in their area of responsibility and with the idea of receiving valuable contributions in terms of the models of business and sustainability of the platform.

#### 4.2.2. Alpha Testing

The alpha testing sessions organized by the task force of the Bilbao City pilot took place during the third and fourth weeks of July 2016. These sessions involved the participation of 20 persons from the different organizations of the task force (University of Deusto, Tecnalia and Bilbao City Council), which tested the platform and the applications from a usability and technical point of view, however, without applying a strict methodology for the user validation.

Tests were focused on identifying problems in three main categories that were critical for the pilot execution: usability, behavior/functional and log generation. In the case of the applications, the in-app questionnaire functionality provided to users was also tested and participants provided their thoughts and feedbacks about the applications using these mechanism.

Participants filled a questionnaire specifically created for this alpha testing phase, which was shared among all of the pilots. In the case of the Bilbao city pilot, they where 18 responses to these questionnaires and the average age of the participants was 34.5. Furthermore, 66.6% of the participants were interested in further collaboration with the project. Participants also identified the most interesting aspects of the platform and those aspects that were not working well and had to be improved. This feedback and the one extracted directly during the event led to the creation of the different issues oriented to improve the applications and the platform during Pilot Phase I.

Table 1 shows the summary of the problems reported in each of the tested categories during the alpha testing sessions. This table also shows the ratio of those problems has been addressed during Pilot Phase I. The table shows that some of the usability problems detected in the applications have not been completely solved during Pilot Phase I. However, the solution of these issues, which are usability issues in their majority, require performing major changes in the application, and therefore, they have been delayed and will be ready for the second pilot phase.

#### 4.2.3. Platform KPI Analysis

This section summarizes the main results obtained after collecting those KPIs for the Bilbao City pilot generated during the Pilot Phase I.

##### KPI1, Open Innovation Area as a Promoter of Public Service Apps

This section present those KPIs related with the usage of the Open Innovation Area as a promoter of public services applications and the co-creation process.

**Figure 8:**
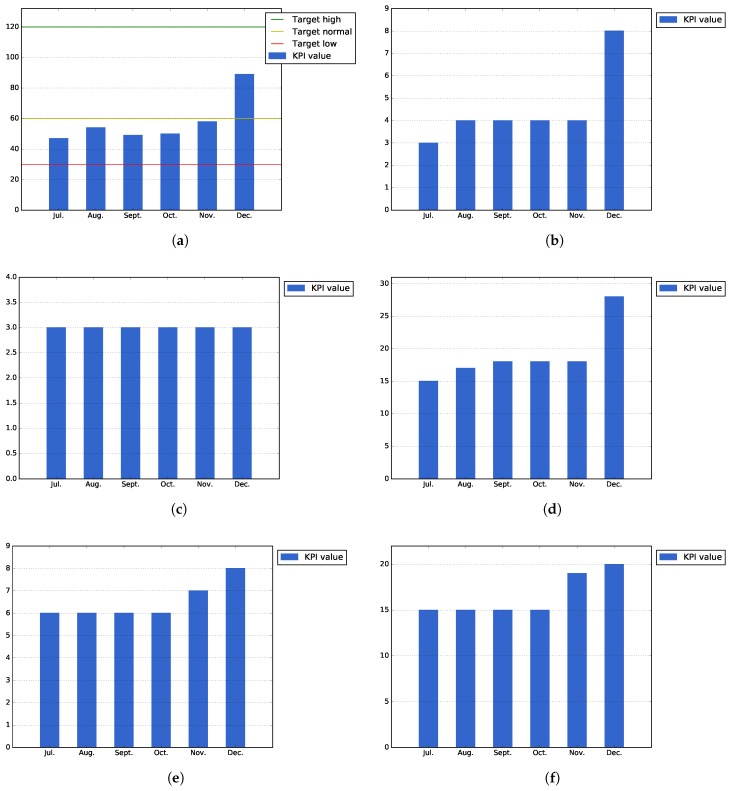
Different KPI values for the Bilbao pilot. (**a**) Number of registered users; (**b**) number of published building blocks; (**c**) number of public services; (**d**) number of ideas; (**e**) number of challenges; (**f**) number of needs.

Figure 8a shows that the total number of registered users into the platform, 89 users, has exceeded the expected normal target for the city of Bilbao, which was established at a number of 60 users. This number of registered users has been achieved thanks to the different events organized in the city: alpha testing phase, specific events with users and the idea contest organized during the month of December.

Figure 8b–d shows the number of actual building blocks, datasets, public services and ideas that have existed at the end of each month during the Pilot Phase I. As can be seen in Figure 8b, some new building blocks were published by the Bilbao city task force during the last part of the execution phase, in order to exemplify the usage of the platform for the incoming idea contests. The number of datasets remains the same (164), as no new datasets have been created in this phase. On the other hand, Figure 8d shows that there has been high increase in the number of published ideas at the end of the pilot due to the same idea contest organized by the Bilbao city council.

On the other hand, Figure 8e,f shows the actual number of challenges at the end of each month, taking into account possible intermediate removals. As shown by both figures, there is an increase of this numbers at the end of the pilot due to the preparation of the idea contest organized by the city council. During the first month of the pilot phase, these numbers have been more stable, as the majority of these challenges were published during the alpha testing and initial events of the task force.

There is another KPI related to the Open Innovation Area, the number of ideas in implementation phase. This KPI remains low, but it is not relevant for the first pilot phase as the objective of this phase was to generate ideas to be implemented during the second phase.

##### KPI2, Open Services Framework as a Facilitator of Public Service App Creation

Those KPIs that are related to the Open Service Framework as a facilitator of the creation of public service applications are summarized in Table 2.

As seen in the table, the values for KPI2.1, during Pilot Phase I, agree with the ones depicted by Figure 8b. The reason is that, during this Pilot Phase I, all of the building blocks are created by the consortium and, therefore, were developed and used to construct the public services developed as part of the work of the Bilbao task force.

On the other hand, KPI2.2 shows that in the case of the Bilbao city pilot, two building blocks were created and were used by other task forces to create their applications, specifically the in-app questionnaire and the query mapper. For the second pilot phase, some of the functionality developed for the applications of the Bilbao city pilot is going to be extracted in order to be available as more reusable blocks that could facilitate the integration and construction of new applications by the rest of the project task forces and, in addition, by the external third party developers.

KPI2.3 and KP2.4 reflect the number of public services created during the Pilot Phase I and the number of those services that are oriented toward promoting the transparency of the administration, respectively. These values have not changed during the pilot phase, as the services were developed internally as part of the task force work, and therefore, they were available since the beginning of the Pilot Phase I. On the other hand, currently only one of the three developed applications (i.e., Bilbozkatu) is oriented toward promoting transparency within the city.

##### KPI3, Stakeholder Behavior Change Towards e-Government

**Figure 9:**
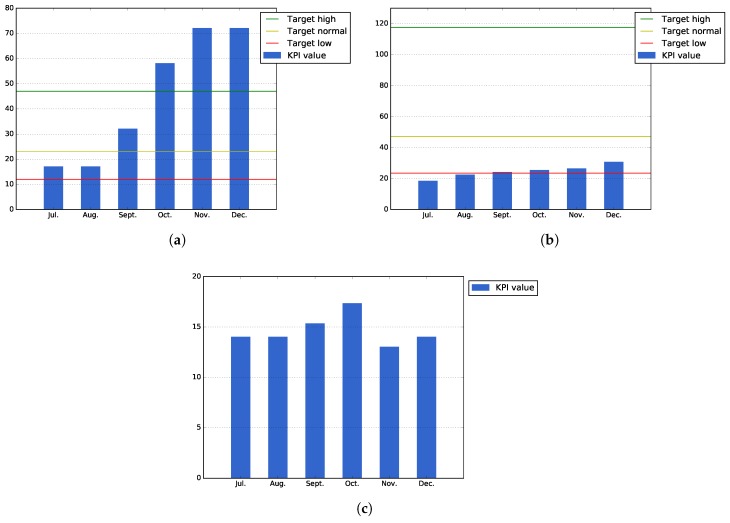
Number of stakeholders participating (**a**), average downloads per application (**b**) and public service usage (**c**) in the Bilbao pilot.

Figure 9a, which measures the accumulated number of attendants that have participated in different events organized during Pilot Phase I, shows that thanks to the events that were organized during the latest months of the period, it has been possible to surpass the expected target values for this KPI.

On the other hand, Figure 9b shows that average downloads of the applications (i.e., total downloads of all applications divided by the number of applications) has surpassed the value that was expected for the low target of this KPI. In order to increase the number of downloads, during the second pilot phase, it will be necessary to perform more events focused on promoting and testing the public services developed during this pilot phase.

Figure 9c shows the public service usage, calculated as the actual installations that the services of Bilbao have at the end each month. As the figure shows, some of the users uninstall the applications after their first usage; this is probably explained by the fact that they think the application is not as useful as they thought. For the second phase, it will be necessary to study, in a more detailed manner, the underlying causes for this user drop-out.

##### KPI4, Age and Gender Distribution of the Registered Users

**Figure 10:**
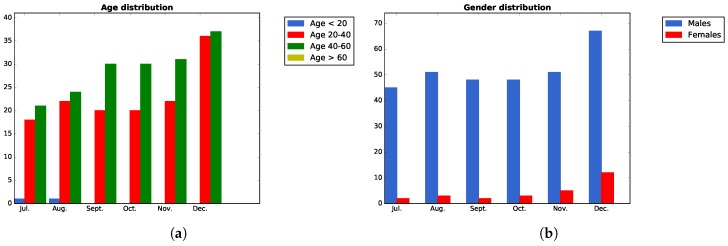
Age (**a**) and gender (**b**) distribution of the registered users in Bilbao.

First, Figure 10a shows that the users that have registered to the platform during this Pilot Phase I belong to a range from 20–40 and from 40–60, with a predominance of the latter over the former. This could be explained by the fact that, in the case of Bilbao, the events were mainly oriented and organized in professional environments. On the other hand, Figure 10b shows that the majority of the participants registered in the platform are males. This can be explained also for the fact, that during Pilot Phase I, the events in Bilbao were mainly oriented to the participation of users from ICT domains.

#### 4.2.4. Public Services Analysis

This section contains the analysis and discussion of those KPIs related with the applications that have been developed and tested in Bilbao during Pilot Phase I: Auzonet, BilbOn and Bilbozkatu.

##### Auzonet

The following sub-sections provide a representation and discussion of the different KPIs that have been collected for the Auzonet application during Pilot Phase I.

**Application KPIs.** Regarding to the number of downloads for Auzonet app, at the end of the first pilot phase, the app was been downloaded 32 times, while the target was 47 downloads. In order to increase this number, it is necessary, during the second phase, to organize a higher number of events focused on promoting the applications. Related to this, we have detected that most users tend to uninstall the application after its usage. During the second phase, it is necessary to study the causes for this user dropout.

At the end of the first pilot phase, the Auzonet application had been started 356 times, exceeding the target value of 150. In addition, 172 users registered into WeLive through the Auzonet application, and it also surpasses the expected value for the application (20 users). However, both KPIs are counting also those events that were generated during the development of the application, and when compared with the actual number of total downloads of the application, it is not possible to obtain such high number of new user registrations.

Therefore, during the second phase, these KPIs should take into account the initial values at the end of the period, and it will be also necessary to remove those events generated during the testing and development period, in order to obtain more representative figures.

**Figure 11:**
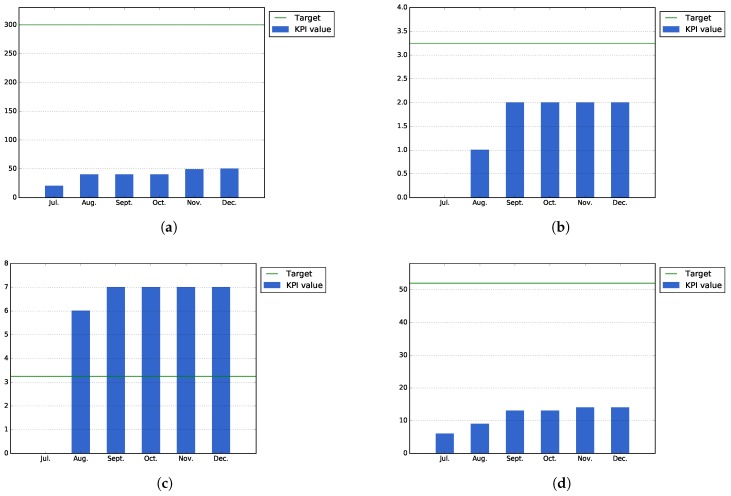
Different KPIs for Auzonet. (**a**) Number of search for item of interest; (**b**) number of borrowed/bought products; (**c**) number of items requested on loan, for sell or donation; (**d**) number of items offered on loan, for sell or donation.

Figure 11a–d represents KPIs designed to measure the usage of the specific functionality of the Auzonet application: the number of searches for items of interest, the number of borrowed/bought products, the number of items requested on load, sell or donation and the number of items offered on loan, for sell or donation, respectively.

Due to the low number of downloads of the application, this number does not provide a real insight of the application usage. However, by observing the usage of the application, it is possible to infer that only a portion of the users are providing products, while the majority of them are searching for or requesting those offered services.

**Figure 12:**
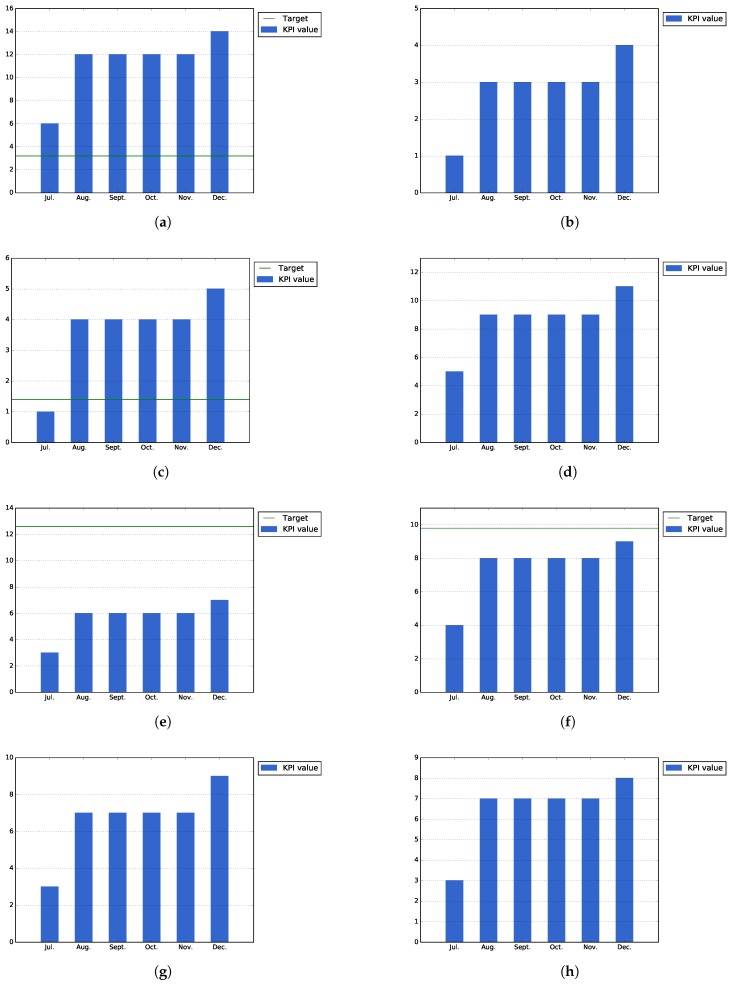
Different KPIs for Auzonet. (**a**) Completed user surveys; (**b**) users who would like to provide detailed feedback; (**c**) users who made suggestions; (**d**) users who think that the app increases their sense of community; (**e**) users who think app is easy to use; (**f**) number of users who consider the app to be useful; (**g**) users who are happy with the design of the app; (**h**) number of users that would recommend the app to a friend.

**In-app questionnaires.** At the end of the first phase pilot phase, the Auzonet application has been downloaded 32 times. As shown in Figure 12a, 14 of those users have filled the in-app questionnaire (43.8% of the total installations). This number exceeds the target expected for the number of filled questionnaires, which was defined as 10% of the total downloads.

On the other hand, Figure 12b shows that only 28.5% of the users that filled out the questionnaire want to be contacted in the future. This value was expected to be low, as the great majority of users do not usually actively participate in further improvements of the application.

Figure 12c shows that 35.7% (five of 14) of the users that filled out the questionnaire has provided improvements for the application. Although this number is higher than the target value (10% of the filled questionnaires), the relative value is expected to decrease when the application is used by other users outside the events organized during Pilot Phase I and, therefore, not highly involved in the application developed.

Finally, Figure 12d shows that 78.6% (11 out of 14 filled questionnaires) of the users that have filled out the questionnaire think that the application increases their sense of being part of a community, which is a good result and one of the main objectives of the application.

According to Figure 12e, at the end of Pilot Phase I, 50.0% of the users that filled out the questionnaire (seven out of 14) thought the application is easy to use. This value is lower than the expected target for this KPI. This means that the design and usability of the application needs to be improved for second phase of the pilots. Nevertheless, the Auzonet app was improved continuously, and as some of the questionnaires were filled out by the users during the initial stages of the application, the results do not reflect all of the changes and updates.

On the other hand, Figure 12f shows that 64.3% (nine out of 14) of the users who filled out the questionnaire think that the application is useful, which is only a bit under the expected target for this KPI established as 70% of the responses.

Figure 12g shows that 64.3% of the users (nine out of 14) liked the application design. However, this value should be taken into account knowing that some users consider that the application still needs some improvements in order to be considered easy to use by the citizens.

Finally, Figure 12h shows that only 57.1% of the users that filled out the in-app questionnaire (eight out of 14) would recommend the application, which is a good value for an application in initial development in its pilot phase.

The main concerns of users related to Auzonet were that the app requested their user and password each time they opened the app and its slowness, since it is implemented as a multi-platform web application instead of a native one. The first concern was solved using the capabilities of the Authentication and Authorization Common Building Block to provide a refresh token, which allows authenticating a user only requesting his/her user and password the first time.

##### BilbOn

The following sub-sections provide a representation and discussion of the different KPIs that have been collected for the BilbOn application during Pilot Phase I.

**Application KPIs.** The total number of downloads during the pilot was 30, not achieving the expected target of this KPI, set at 47 downloads. In addition, there was a drop-out of users after their installation of the application, reducing the current number of downloads to 15. Therefore, during the second pilot phase, it is necessary to increase the number of events oriented toward promoting the BilbOn application and to investigate the causes that make users uninstall the application after its usage.

On the other hand, the application has achieved the desired target for its number of executions (172 out of 150) and surpassed the expected number of registered users through the application (38 out of 20). However, as occurred with the Auzonet application, due to the low number of installations, these numbers are mainly explained by tests done during its development and other tests.

**Figure 13:**
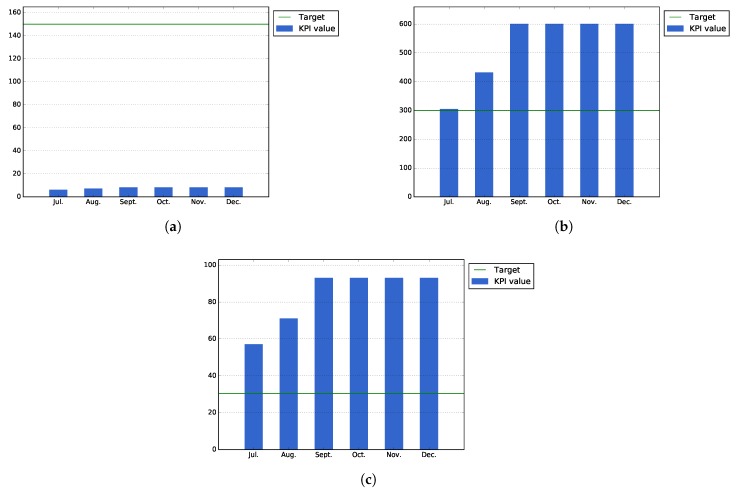
Different KPIs for BilbOn. (**a**) New POIs added; (**b**) searched POIs; (**c**) selected POIs to view details.

As Figure 13a–c shows, users tend to use the application as consumers, searching or obtaining information about the available POIs, and only a small number of events is related to the inclusion of new information by the user. However, these figures should be taken cautiously due to the low number of installations, meaning that they are not representative enough of the real usage of the application.

**In-app questionnaires.** The following figures show the historic values of those KPIs that are obtained from the in-app questionnaire for the BilbOn application.

**Figure 14:**
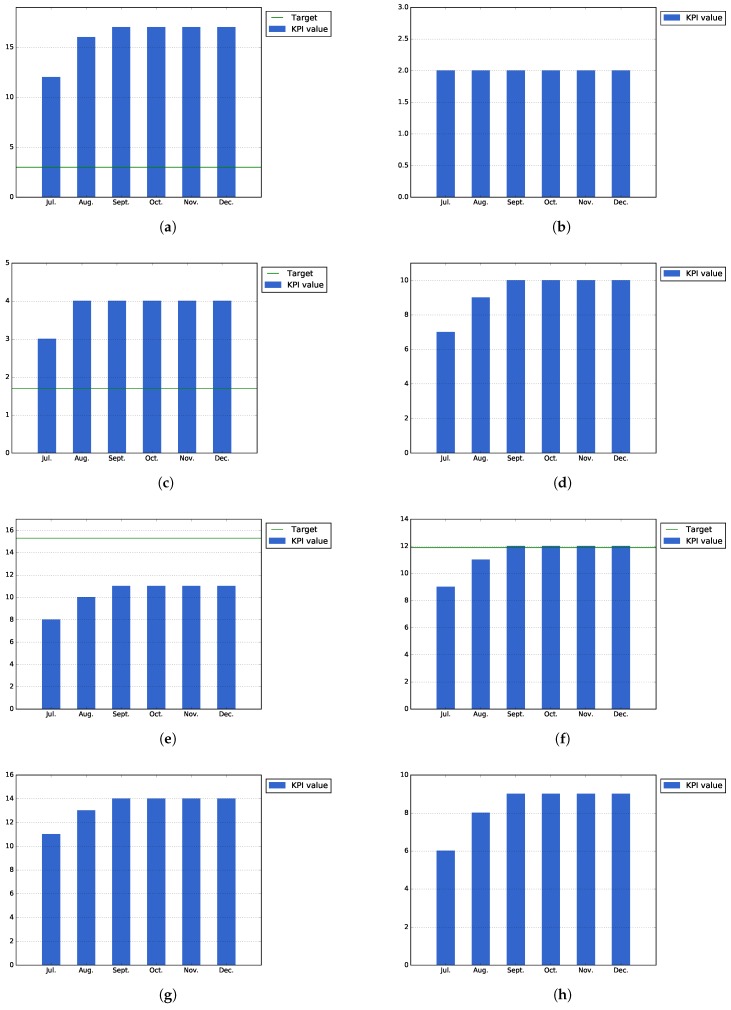
Different KPIs for BilbOn. (**a**) Completed user surveys; (**b**) users who would like to provide detailed feedback; (**c**) users who made suggestions; (**d**) users who think that the app increases their sense of community; (**e**) users who think the app is easy to use; (**f**) users who consider the app to be useful; (**g**) users who are happy with the design of the app; (**h**) users that would recommend the app to a friend.

In the first place, Figure 14a shows that the expected target for the number of completed in-app questionnaires has been exceeded in the case of the BilbOn application. At the end of Pilot Phase I, 17 questionnaires have been filled out through the BilbOn application. This target was defined as a portion (10%) of the total downloads of the application, which are 30 downloads for BilbOn at the end of Pilot Phase I.

On the other hand, Figure 14b shows that only 11.8% (two out of 17) of the users would like to be further contacted for further questioning.

Figure 14c shows that 23.5% of users (four out of 17) have provided suggestions for the application; while, as Figure 14d shows, 58.8% of the users (10 out of 17) think that the application increases their sense of community.

In addition to the previous KPIs, Figure 14e shows that only 64.7% of the users that have filled out the questionnaire (11 out 17) think that BilbOn is easy to use, which is under the expected value for this KPI, established at 90% of the filled out questionnaires. On the other hand, 70.5% of the responses (seven out of 17) think that the application is useful (Figure 14f), which is just at the specified target for this KPI.

Finally, 82.3% of the users (14 out of 17) are happy with the design of the app, as shown by Figure 14g, while 52.9% (nine out of 17) would recommend the application to a friend (Figure 14h).

The main issues detected by users for BilbOn were about the quality and integrity of the data (some of them thought that it should be checked by some administrator before publishing it) and different usability issues.

##### Bilbozkatu

**Application KPIs:** The Bilbozkatu application was downloaded 30 times, which is under the expected number of downloads (47). In addition, the number of users that maintain the application installed in their devices decreased after some time, to 13 users. Therefore, it is necessary, during the second pilot phase of the project, to increase the number of events oriented toward the use of the application, in order to achieve a critical mass of citizens that install and use the application.

The number of executions of Bilbozkatu at the end of the testing period exceeds the expected target for this KPI (180 out of 150). In addition, the number of users that have registered into WeLive through the Bilbozkatu application also surpasses the expected value for the application (36 out of 20). However, both KPIs are counting also those events that were generated during the development of the application, and when compared with the actual number of total downloads of the application, it is not possible to obtain such a high number of new user registrations.

Therefore, during the second phase, these KPIs should take into account the initial values at the end of the period, and it will be also necessary to remove those events generated during the testing and developed period, in order to obtain more representative figures.

**Figure 15:**
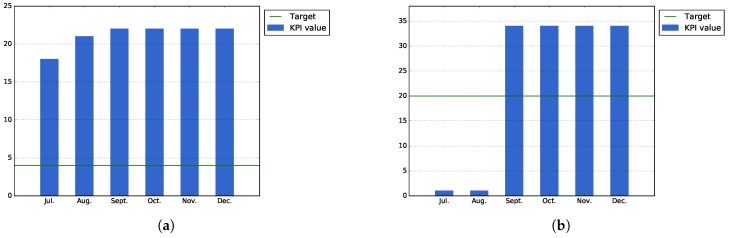
Different KPIs for Bilbozkatu. (**a**) Voting campaigns organized; (**b**) votes per campaign received.

Figure 15a,b shows the values for those KPIS that are specific for the Bilbozkatu application, the number of voting campaigns organized and the number of votes received per campaign, respectively. As shown by the figures, both values exceed the expected target value; however, as in the previous cases, it should be taken into account that these values could not be representative enough of the usage of the application, due to the low number of downloads and usage that the Bilbozkatu application had during the Pilot Phase I.

**In-app questionnaires:** The following figures show the historical values of those KPIs that are obtained from the in-app questionnaire for the Bilbozkatu application.

**Figure 16:**
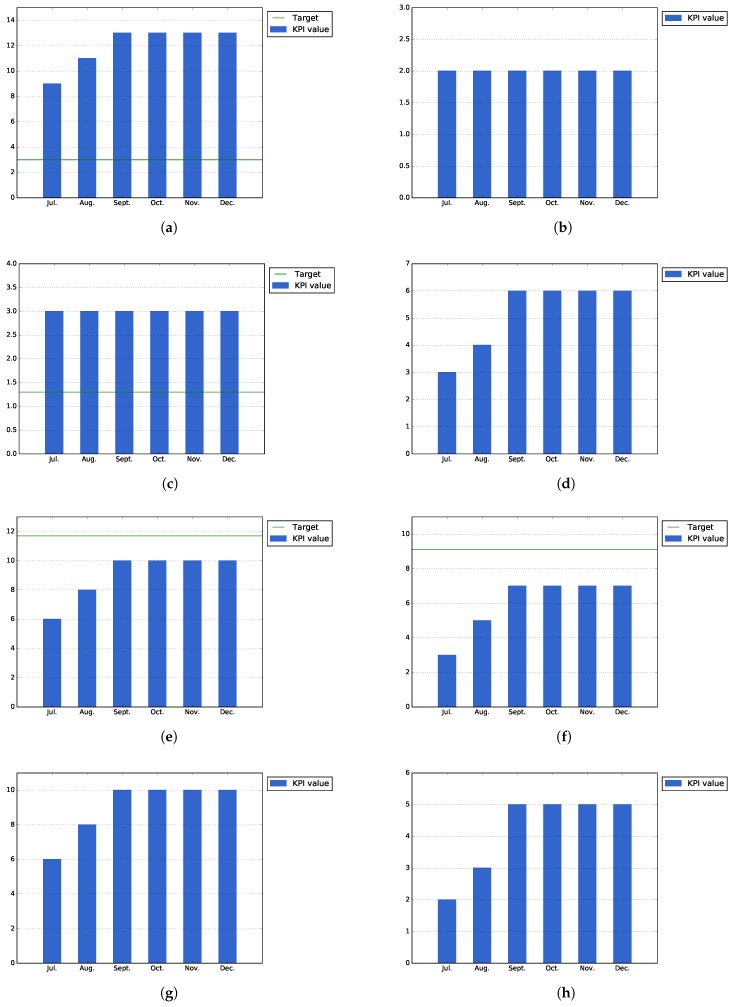
Different KPIs for Bilbozkatu. (**a**) Completed user surveys; (**b**) users who would like to provide detailed feedback; (**c**) users who made suggestions; (**d**) users who think that the app increases their sense of community; (**e**) users who think the app is easy to use; (**f**) users who consider the app to be useful; (**g**) users who are happy with the design of the app; (**h**) users that would recommend the app to a friend.

In the first place, Figure 16a shows that the expected target for the number of completed in-app questionnaires has been exceeded in the case of the Bilbozkatu application. At the end of Pilot Phase I, 13 questionnaires have been filled out through the Bilbozkatu application. This target was defined as a portion (10%) of the total downloads of the application, which are 30 downloads for Bilbozkatu at the end of Pilot Phase I.

On the other hand, Figure 16b shows that only 15.3% (two out of 13) of the users would like to be further contacted for further questioning.

Figure 16c shows that 23% of users (three out of 13) have provided suggestions for the application; while as Figure 16d shows, 46.15% of the users (six out of 13) think that the application increases their sense of community.

In addition to the previous KPIs, Figure 16e shows that only 76.9% of the users that have filled out the questionnaire (10 out 17) think that BilbOn is easy to use, which is under the expected value for this KPI, established at 90% of the filled questionnaires. On the other hand, 53.8% of the responses (seen out of 13) think that the application is useful (Figure 16f), which is just at the specified target for this KPI.

Finally, 76.9% of the users (10 out of 13) are happy with the design of the app, as shown by Figure 16g, while 38.4% (five out of 13) would recommend the application to a friend (Figure 16h).

The main concerns of the users regarding Bilbozkatu were about the number of categories to classify their proposals and ideas and that the decisions made by citizens should be binding. Bilbao City Council claimed that this was not the aim of the app and that binding decisions could have serious political implications.

#### 4.2.5. Demographic Questionnaires

**Figure 17:**
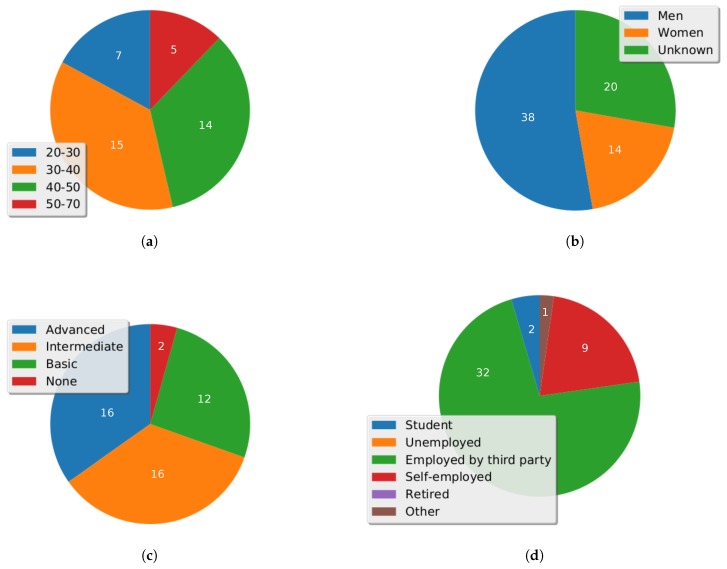
Age (**a**), gender (**b**), ICT level (**c**) and work status (**d**) distribution of attendants to the Bilbao events.

There has been a total attendance of 72 persons to the different organized events, with the following distribution per gender and age, according to Figure 17a,b. Regarding the gender, the “Unknown” value represents participants that did not wanted to reveal their gender.

On the other hand, Figure 17c,d shows, taking into account all of the organized events, a summary of the audience ICT level and their works status, respectively.

#### 4.2.6. User Support

During Pilot Phase I, the users have been provided with a contact e-mail address for support about the usage of the platform and the applications. For the Bilbao pilot, eight requests were done by e-mail, answering successfully 100% of them.

#### 4.2.7. Results from the Novi Sad, Trento and Helsinki-Uusimaa Pilots

In addition to the results extracted from the Bilbao pilot, we expose some conclusions obtained from the Novi Sad, Trento and Helsinki Uusima pilots. More centered on the technical and functional aspects of the platform and apps, we can conclude from the Novi Sad pilot that the WeLive platform allowed the city council to define challenges in order to obtain feedback from citizens and application developers and to publish the existing Novi Sad open data as datasets. Citizens from Novi Sad were able to publish their ideas using the Open Innovation Area, although some users complained that the user interface was too complex. The number of users of the applications was low; thus, the Novi Sad pilot has to put more effort into promoting the developed apps during the second pilot phase.

In the case of Trento, the most important concern of citizens was the mixing of different pilots within the platform. Users demand a clearer distinction among different city pilots in the platform. In spite of this, the pilot in Trento was successfully executed, with a proper implication of the citizenship and the city council. Regarding the developed apps, citizens demand more functionalities. For implementing these new functionalities, the city council must publish new datasets.

The evaluation results produced by Helsinki-Uusimaa are focused mainly on the usability of the platform. Users from this region complain about its complexity and the lack of usability of some tools.

## 5. Related Work

According to [12], novel approaches and processes are required with a cross-government, decentralized and multi-actor architecture, coupled with the role of social tools and integration of big data. For enabling a new user-centric collaborative public service ecosystem, the ability of sharing, interacting and collaborating among different actors must be exploited, resulting in new types of infrastructure where public value is created. These new infrastructures must tackle potentials problems related to security, privacy, data protection and interoperability (at the technological and organizational level) issues; taking advantage of open standards, adoption of linked data principles and cloud computing, that is development 3.0.

As research demonstrates the potential of mobile apps to provide access to public information and to transform governments, m-Government (mobile app-engaged Government) must be the next trend for ICT use in public administrations [13].

Open Innovation [14] is a co-creation process that facilitates different stakeholder engagement (i.e., quadruple helix) where governments and companies should use external ideas from citizens or academia to develop their services in a collaborative Public Private Partnership setting. In summary, it provides a framework for involving actual customers in the product and service innovation process. Increasing the focus on development and sustainability of smart cities, a more inclusive public sector can be enabled, i.e., one that incorporates different stakeholders into the planning of development activities of the city through multiple channels, enabling their engagement on various matters. Living Labs [15] is one of the results of this approach, a real environment where new services for cities can be deployed and tested, capturing the contributions and feedback given by the citizens.

The WeLive project is devised to transform the current e-government approach followed by most public administrations into we-government [16] where all of the stakeholders of public administration, namely citizens, local businesses and companies, are treated as peers (collaborators) and prosumers (providers) instead of the usual customer role associated with them. Furthermore, this project also has ambitions to turn e-government into t-Government (transformational Government) [17] by providing the public with the technology tools that enable them to create public value themselves. In addition, WeLive is also thought to embrace l-Government (lean Government) [18] that aims to do more with less by involving other players, leaving the government as an orchestrator around enabled platforms.

Among projects related to WeLive, we can found the following ones:

- SmartCampus (http://www.smartcampuslab.it/) is a project that aims at catalyzing the creativity and enthusiasm of all people and institutions of the university campus and to produce innovative services designed to support everyday individual and social life and ease the operation of the campus. The technological backbone of SmartCampus is an ICT platform for participatory service design and delivery.
- FI-WARE –Future Internet-ware– (http://www.fi-ware.org/) is an innovative, open cloud-based infrastructure for cost-effective creation and delivery of Future Internet applications and services.
- The main goal of the IES Cities –Internet Enabled Services for the Cities across Europe– (http://iescities.eu) project is to facilitate the use of an open technological platform in different cities across Europe, allowing the citizens providing and consuming internet-based services based on their own and external linked data related to the cities. As a result of the project, an open platform for the generation of user-oriented Internet services making use of the data facilitated by the users’ smartphones and from the different cities sources (sensors and open data) was made available. The added value, compared to other initiatives, was obtained thanks to the participation of the users, which is considered one of the key remarkable factors in the proposal.
- The SIMPATICO –SIMplifying the interaction with Public Administration Through Information technology for Citizens and cOmpanies– (http://www.simpatico-project.eu/) project (in development) aims to improve the experience of citizens and companies in their daily interactions with the public administration by providing a personalized delivery of e-services based on advanced cognitive system technologies. The goal will be achieved through a solution based on the interplay of language processing, machine learning and the wisdom of the crowd (represented by citizens, business organizations and civil servants) to change for the better the way citizens interact with the Public Administration.
- MUGGES –Mobile User Generated GEo Services– (http://www.future-internet.eu/activities/fp7-projects.html) is an FP7 project that tested the super-prosumer platform components implemented in 7FP-mCiudad. MUGGES trialled the mobile super-prosumer concept with real users of location-based services.
- ADAPTA –Adecuación, validación e integración de datos abiertos por gobiernos y empresas– (https://morelab.deusto.es/projects/info/adapta/) is a Spanish research project that defined, adapted and integrated a tool set allowing the tuning, validation and integration of open data by governments, businesses and citizens.
- SocIoTal –Socially aware and citizen-centric Internet of Things– (http://sociotal.eu/) is an FP7 project that aims at creating an all-inclusive Internet of Things infrastructure for the society by accelerating the creation of a socially-aware citizen-centric Internet of Things. The project is focusing on defining tools for co-creation of IoT services that are of interest to citizens.
- The FP7 IoT Lab (http://www.iotlab.eu/) is an FP7 project focused on the creation of a platform that will enable crowdsourcing mobile devices suitable for execution of experiments or applications. The goal is to investigate social and economic incentives that will drive the owners of devices to make their devices available for execution of experiments and applications.
- The Helsinki Region Infoshare (HRI) (http://www.hri.fi/en/about/) initiative aims to make regional information quickly and easily accessible to all. HRI is a web service for fast and easy access to open data sources between the cities of Helsinki, Espoo, Vantaa and Kauniainen. The data published are mainly statistical, giving a comprehensive and diverse outlook on different urban phenomena, such as living conditions, economics and well-being, employment and transport.
- Do-it-Yourself Smart Experiences (https://itea3.org/project/diy-smart-experiences.html): The ITEA2 –Information Technology and European Advancement– Do-it-Yourself Smart Experiences supports users in the Creating Aware, Interactive and Flowing Experiences In An Internet-Of-Things World.
- The CoCo –from Co-production to co-creation– Toolkit (http://www.openlivinglabs.eu/news/coco-toolkit-adapting-co-creation-activities) project enables businesses to adopt a co-creative business approach, evaluate and communicate their current business approach and co-create together with different stakeholders.
- The Service Innovation through Strategic Stakeholder Integration (SISSI) (http://www.hanken.fi/public/en/sissi) project aims to benchmark, test and develop tools, practices and methods for involving stakeholders. It develops models enabling effective participatory stakeholder integration for the purpose of enhancing knowledge and innovative practices adaptable for Finnish companies.
- MassIdea (http://www.massidea.org/) is a Finish project, which developed a free of charge open innovation community platform where people can share their ideas, discuss today’s challenges, as well as visions of the future; key factors when creating new innovations. Technologically, massidea.org is grounded on open source solution.
- CLIPS –Cloud approach for Innovation in Public Services– (http://ec.europa.eu/digital-agenda/en/clips-project-cloud-public-services) is a CIP-ICT-PSP pilot action aimed at the development of a new approach to the delivery of public services through the use of cloud computing. It will provide a framework that can be used for the evolution of cloud-based public services that seeks to overcome some of the major issues associated with cloud uptake within the public sector notably in architecture, design and trust. The definition and the implementation of new services follows a mash-up approach, to develop an ecosystem template (methodology and toolkit) that involves all of the possible stakeholders (i.e., civil servants, public authorities, citizens and businesses) and where services are defined specifically for the benefit of a municipality, which can be replicated across Europe.
- X-Net.Lab –eXtended Net.Lab– (http://www.opencoesione.gov.it/progetti/1misedm232722/) aimed at creating a platform, named PLAtform and TOols for Networked E-business (PLATONE), that enables the identification of networked enterprises by means of digital business ecosystem paradigms. Actors of DBEsare enterprises, according to the new “Enterprise 2.0” paradigm.

While these projects focus on the implementation of a particular area of the Open Government paradigm (Open Data generation and publication, idea or business co-creation, delivery of public services, and so on), the main advantage of WeLive over them is that it encompasses multiple areas together (collaborative research and development, Open Data management, services and building block publication, user metadata management and service customization) as exposed in Section 2.

The idea of Open Government has been disseminated around the world quickly, since the first public administrations started sharing their data as Open Data. Europe leads the region ranking [19], with widely known initiatives like data.gov.uk (U.K.), opendata.dk (Denmark) or data.gouv.fr (France). However, many countries seriously lack the potential to exploit Open Data in order to adopt Open Government policies. While many of them put many efforts into implementing open data portals, they are placing little efforts into transforming open data into a real value, bringing them closer to citizens and entrepreneurs through APIs to be consumed by application developers [20], targeted by WeLive.

## 6. Conclusions and Further Work

This paper has presented the WeLive platform tools and its co-creation approach applied in order to give place to the first generation of WeLive compliant apps. The evaluation methodology designed to be applied in two phase pilots has also been outlined.

At this first pilot phase, the WeLive platform has enabled Bilbao’s city council to define challenges in order to obtain feedback from citizens and application developers and users to publish their ideas using the functionality provided by the platform. However, users complained that the user interface of some of the components was too complex. To solve problems with the user interface, it is planned to use a new version of the UI that has focused on easing the user interaction with the platform during the second pilot phase. This improved UI is being tested using techniques like the “think aloud” technique [21].

Although the number of total registered users has exceeded the expected target for Pilot Phase I, the activity of these users in the platform is not as high as expected, and it has had a high user drop-out in the usage of the applications. For the second phase, it is necessary to increase the number of events promoting both the platform and the applications.

In addition to the platform, apps created during the “Stakeholders consultation process” have been tested. Although these apps have received good feedback about their functionality and future usage from users participating in testing events, the number of users of these applications shows that maybe participants from focus groups do not form a representative set of citizens from Bilbao or a proper dissemination has not been done. For Pilot Phase II, it is necessary to promote the usage of these apps through a strong advertising campaign with the objective of extracting more feedback from the users.

During the second pilot phase, to be held in the second half of 2017, in addition to the functionalities of the WeLive platform tested during the first phase, the development of the building blocks and apps by users will be encouraged. We want to experiment not only with the co-ideation of services across stakeholders, but also with the co-implementation of services, so that the full co-creation process proposed by WeLive is evaluated. Further, in the second pilot phase, the usage of the Visual Composer, a component that enables creating mash-ups among different building blocks and open datasets hosted at the WeLive Marketplace, will be launched and promoted.

## Figures and Tables

**Table 1:** Issues reported for the applications during the alpha testing sessions in Bilbao.

Application	Usability			Behavior			Log Generation			In-App Questionnaire		
**Auzonet**	Total	13	-	Total	4	-	Total	0	-	Total	1	-
	Open	5	38.46%	Open	1	25%	Open	0	-	Open	0	0%
	Closed	8	61.54%	Closed	3	75%	Closed	0	-	Closed	1	100%
**BilbOn**	Total	7	-	Total	1	-	Total	1	-	Total	1	-
	Open	4	57.14%	Open	0	0%	Open	0	0%	Open	0	0%
	Closed	3	42.86%	Closed	1	100%	Closed	1	100%	Closed	1	100%
**Bilbozkatu**	Total	9	-	Total	4	-	Total	1	-	Total	1	-
	Open	5	55.56%	Open	0	0%	Open	0	0%	Open	0	0%
	Closed	4	44.44%	Closed	4	100%	Closed	1	100%	Closed	1	100%
**WeLive Player**	Total	9	-	Total	4	-	Total	1	-	Total	1	-
	Open	5	55.56%	Open	0	0%	Open	0	0%	Open	0	0%
	Closed	4	44.44%	Closed	4	100%	Closed	1	100%	Closed	1	100%

**Table 2:** Summary of KPIs related to the OSF as a facilitator of public service creation in Bilbao.

KPI	July	August	September	2016 October	November	December	High	Target Normal	Low
KPI2.1, Number of building blocks created with the help of the Open Service framework and Open Data	5	6	6	6	6	8	**19**	**9**	**5**
KPI2.2, Number of building blocks and apps exchanged among pilot cities	2	2	2	2	2	2	**5**	**3**	**2**
KPI2.3, Number of public service apps created with the Open Services Framework	3	3	3	3	3	3	**9**	**8**	**4**
KPI2.4, Number of public service apps promoting transparency	1	1	1	1	1	1	**5**	**4**	**4**

## Abbreviations

The following abbreviations are used in this manuscript:

AAC	Authentication and Authorization Control
ADS	Analytics Dashboard
API	Application Programming Interface
BB	Building Block
CIP	Competitiveness and Innovation Framework Programme
CRUD	Create, Read, Update and Delete
CSV	Comma Separated Values
DCAT	Data Catalog vocabulary
EU	European Union
FAQ	Frequently Asked Questions
FP7	Seventh Framework Programme
GDP	Gross Domestic Product
HTTP	HyperText Transfer Protocol
ICT	Information and Communications Technology
IoT	Internet of Things
ITEA	Information Technology for European Advancement
JSON	JavaScript Object Notation
KPI	Key Performance Indicator
L-USDL	Linked Unified Service Description Language
PA	Public Administration
POI	Point of Interest
PSP	Policy Support Programme
ODS	Open Data Stack
RDF	Resource Description Format
REST	Representational State Transfer
UI	User Interface
URL	Uniform Resource Locator
XML	eXtensible Markup Language
WADL	Web Application Description Language
